# Data-Driven Modelling and Control for Robot Needle Insertion in Deep Anterior Lamellar Keratoplasty

**DOI:** 10.1109/lra.2022.3140458

**Published:** 2022-01-05

**Authors:** William Edwards, Gao Tang, Yuan Tian, Mark Draelos, Joseph Izatt, Anthony Kuo, Kris Hauser

**Affiliations:** Department of Computer Science, University of Illinois at Urbana-Champaign, Urbana, IL 61801 USA; Department of Computer Science, University of Illinois at Urbana-Champaign, Urbana, IL 61801 USA; Department of Biomedical Engineering, Duke University, Durham, NC 27708 USA; Department of Biomedical Engineering, Duke University, Durham, NC 27708 USA; Department of Biomedical Engineering, Duke University, Durham, NC 27708 USA; Department of Ophthalmology, Duke University, Durham, NC 27710 USA; Department of Computer Science, University of Illinois at Urbana-Champaign, Urbana, IL 61801 USA

**Keywords:** Medical robots and systems, surgical robotics: planning, model learning for control

## Abstract

Deep anterior lamellar keratoplasty (DALK) is a technique for cornea transplantation which is associated with reduced patient morbidity. DALK has been explored as a potential application of robot microsurgery because the small scales, fine control requirements, and difficulty of visualization make it very challenging for human surgeons to perform. We address the problem of modelling the small scale interactions between the surgical tool and the cornea tissue to improve the accuracy of needle insertion, since accurate placement within 5% of target depth has been associated with more reliable clinical outcomes. We develop a data-driven autoregressive dynamic model of the tool-tissue interaction and a model predictive controller to guide robot needle insertion. In an *ex vivo* model, our controller significantly improves the accuracy of needle positioning by more than 40% compared to prior methods.

## I. Introduction

DEEP anterior lamellar keratoplasty (DALK) is a cornea transplantation technique which has been shown to improve patient outcomes compared to prior methods. Where as the alternative penetrating keratoplasty (PKP) method transplants the full thickness of the cornea, DALK is a partial thickness transplant, replacing only the anterior layers (around 90% of the total thickness), while leaving the original endothelium and Descemet’s membrane intact [1]. This has been shown to significantly reduce the risk of tissue rejection [2].

**Fig. 1.**
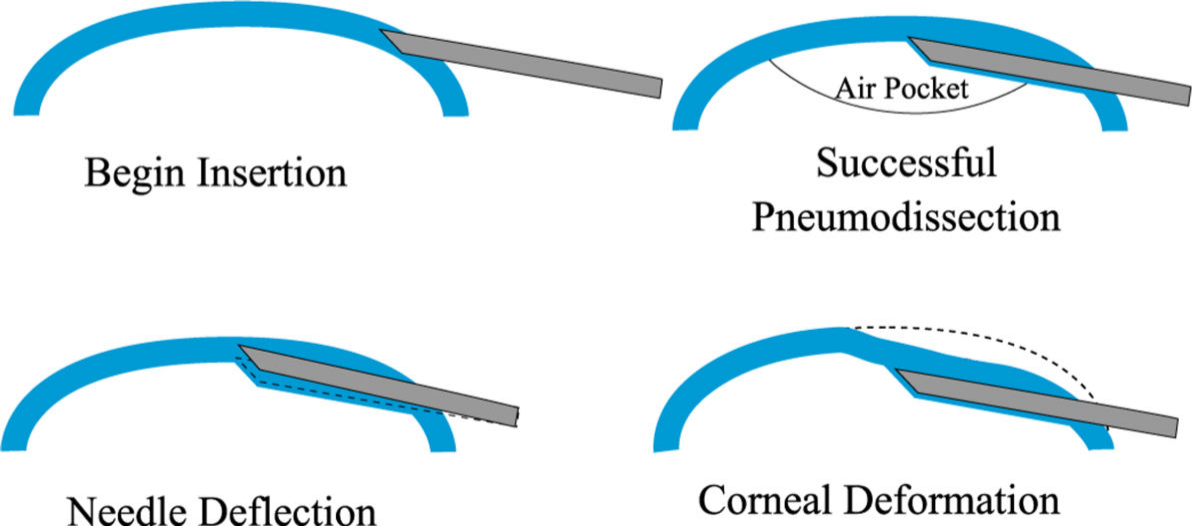
Top: In deep anterior lamellar keratoplasty (DALK), a cannulation needle is inserted from the side of the cornea and air is injected to separate the cornea layers for dissection. Bottom: Needle deflection and corneal deformation are sources of error that can prevent the needle from reaching the desired target depth.

One approach to DALK is the “big-bubble” technique. As shown in Fig. 1, a cannulation needle is carefully inserted into the cornea and its tip is positioned just above the cornea apex. Air is then injected through the needle to perform pneumodissection. Ideally, an air bubble is formed which separates the endothelium and Descemet’s membrane from the anterior cornea layers [3]. In practice, the air bubble frequently fails to form properly. Borderie *et al.* [4] found that the bubble failed to achieve pneumodissection in around 59% of cases, though the success rate can vary significantly depending on the surgeon and technique [5].

Successful bubbles can be classified as either type I or type II. Type I bubbles form within the stroma, while type II bubbles form deeper, between the stroma and the Descemet’s Membrane. Depending on the type of bubble formed, the surgeon will have to modify the graft preparation [6]. Moreover, the type of bubble impacts the perforation rate and the histological properties of the graft [6], [7]. Thus, it is highly desirable to control the type of bubble formation. Yoo *et al.* [6] found that the relative depth of the needle within the cornea is an important factor for both the success rate and type of bubble formation. They found that type I bubbles were consistently formed when the relative depth of the needle was between 75% and 85%, while type II bubbles dominantly formed for depths greater than 90%. Depths between 85% and 90% yielded a mix of bubble types. Thus, when the needle depth can be controlled to within 5% of intended, it is possible to reliably achieve bubble formation of a particular type. Depending on thickness of the cornea, this corresponds to an accuracy of approximately 30 *μ*m, which is challenging for human surgeons to achieve due to the small scales and difficulty of depth perception [8].

The need for small scale manipulation and visualization make the DALK procedure a promising application for surgical robotics, and there have been several recent works addressing this. Guo *et al.* [9] and Park *et al.* [10] use custom robots to guide the needle insertion, but these devices have limited range of movement and are limited to steeper needle insertion angles, which has been associated with worse pneumodissection outcomes [11]. Draelos *et al.* [12], [13] develop a surgical robot system which mounts the needle on a 6-DoF arm, granting a wider range of movement. The needle insertion is guided using feedback from an optical coherence tomography (OCT) sensor, which images the location of the needle with respect to the cornea surfaces. The system can be operated either in cooperative mode, wherein the needle is guided by a human surgeon, or automatic mode, where in the needle insertion is done autonomously.

**Fig. 2.**
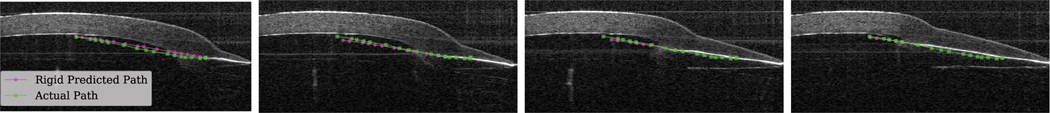
Four points in time of the same needle insertion. Notice that the rigid predicted path (magenta) diverges from the actual path (green) and that the cornea surface deforms as the insertion proceeds.

Planning for automatic needle insertion in Draelos *et al.* is based on the assumption that both the cornea and needle remain rigid [13]. However, they observe that as the needle is inserted into the cornea, the relationship between the robot end-effector and the needle tip deviates from this rigid assumption, suggesting that either the needle or some component of the attachment assembly is deforming. Moreover, the cornea itself can deform significantly as the needle is inserted, effectively creating a moving target. Fig. 1 illustrates both of these effects and Fig. 2 shows examples in OCT data. To address these issues, the authors’ approach reacts to deformation via feedback control rather than constructing an accurate plan. Once embedded, the needle cannot be translated laterally, which makes it difficult to correct course once the needle has drifted off track. This can cause the needle to miss its target position by more than 100 *μ*m.

This paper presents an insertion controller which accounts for the predicted needle and cornea deformation. A data-driven model is created to predict the motion of both the needle and the cornea in response to the robot’s motion. A model predictive controller (MPC) then uses this model to plan an insertion path consistent with the learned dynamics. We use a *cross-simulation controller selection* procedure, similar to cross-validation, to compare candidate models for MPC without using physical experiments.

An autoregressive linear model (ARX) is chosen for the MPC controller based on this scoring procedure. We evaluate the method on *ex vivo* corneas and find that it outperforms the state-of-the-art [13] in terms of both final error in cornea depth achieved by the needle (3.96 ± 1.48% vs 7.80 ± 3.37% for a target depth of 90%) and in terms of vertical error (43.29 ± 15.78 *μ*m vs 75.71 ± 33.90 *μ*m). Furthermore, the method achieves less than 5% error in relative depth (the threshold found by Yoo *et al.* [6] to be associated with reliable control of bubble type) in 61% of trials, as opposed to 42% for the baseline.

## II. Related Work

Robots have been widely studied in microsurgery applications to overcome the limits of human perception and dexterity. Tasks to which surgical robots have been applied include cochlear implantation [14], vascular anastomosis [15], and variocolectomy [16]. In particular, there has been considerable effort to develop robotic surgical tools for ophthalmic surgery [17]–[20], including systems which use magnetic fields to guide the tool [21], [22]. Many of these systems are designed for teleoperation by human surgeons, but there has also been work on automatic motion planning for surgical robotics. In particular, automatic steering of flexible needles has been done using sampling-based planners [23] and inverse kinematics [24], though these techniques require a model of needle-tissue interaction. Learning from demonstration (LfD) has also been used for surgical tasks [25], [26], and Keller *et al.* [27] uses LfD and reinforcement learning (RL) for DALK. LfD and RL can pose safety concerns in a surgical setting and typically require large amounts of data to be effective, which can be expensive to obtain.

A significant challenge in surgical robotics is modelling the interaction of surgical instruments and tissues [28]. Finite Element Modelling (FEM) has been used to model such interactions [29], but such FEM models are often difficult to develop and computationally intensive to simulate. They are, therefore, not well-suited for the real-time demands of surgical robots. In other robotics domains, data-driven model predictive control (MPC) has been used to control challenging systems such as cutting food [30], aggressive driving [31], and robotic fish [32]. Data-driven MPC is less computationally intensive than techniques like FEM and more data-efficient than LfD and RL, making it a more suitable choice for surgical robotics. In this work, we apply data-driven MPC to the DALK task.

## III. DALK Workstation

**Fig. 3.**
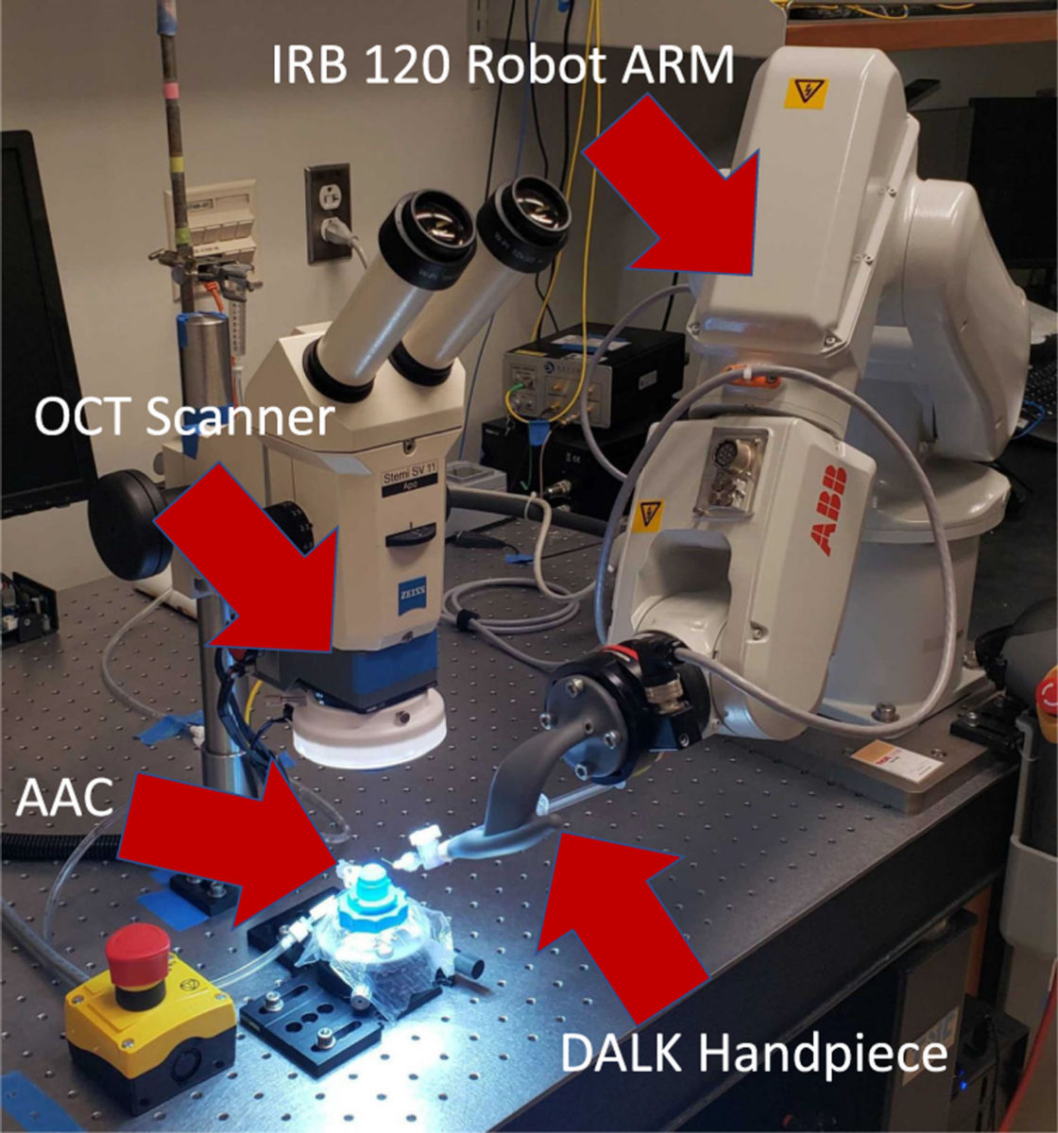
The DALK Workstation uses an IRB 120 robot arm which holds the needle via the DALK Handpiece. The artificial anterior chamber (AAC) holds the cornea sample while the OCT scanner provides feedback.

Our experimental platform is the DALK workstation (shown in Fig. 3) previously described in [13]. It includes a manipulation subsystem, which guides the needle, and a perception subsystem, which tracks the position of the needle and cornea. The manipulation subsystem consists of an IRB 120 robot arm (ABB robotics; Shanghai, China), and a custom-designed “DALK handpiece,” which attaches to the robot’s end-effector and holds the 27-gauge cannulation needle. The arm and handpiece are designed so that they can guide the needle into the cornea without colliding with either the patient’s anatomy or other equipment in the surgical theater. The perception subsystem consists of a custom optical coherence tomography (OCT) scanner mounted beneath a stereo microscope. The OCT scanner captures volumetric images of the cornea and needle during the DALK procedure at a rate of ~1.5 Hz. Fig. 2 shows sample cross-sections of the OCT volumetric image, capturing the needle at several points through the insertion.

The perception subsystem extracts features of interest from the OCT-acquired volumetric image in real-time. The anterior and posterior cornea surfaces are segmented using Dijkstra’s algorithm using the method described in [33]. The cornea apex is then identified by fitting a parabola to the posterior cornea surface. The needle is tracked by matching a 3D model of the needle to the OCT voxels using the iterative closest point algorithm [34]. The positions of both the needle and the posterior cornea surface are corrected for the index of refraction imposed by the curved anterior cornea surface. Refer to [13] for more details.

Draelos *et al.* [13] also proposed methods for automatic needle insertion. The insertion is divided into an *embedding phase* and an *advancement phase*. During the embedding phase, the robot executes an open-loop motion to embed the needle tip in the peripheral cornea. During the advancement phase, the needle is steadily advanced centripetally. Ideally, the needle tip moves steadily deeper as it is advanced, reaching the target depth at the cornea apex. A target depth of 90% is used since this depth has been associated with successful pneumodissection [35]. The advancement phase uses feedback from the perception system to correct the needle if it drifts off path. Draelos *et al.* describe multiple advancement planners; however, we take as a baseline the *line planner*, which attempts to follow a straight line path between the needle tip and the target position under the assumption of no needle or cornea deformation. In this work, we design a data-driven model predictive controller for the advancement phase which predicts and plans for the mechanical effects of needle-cornea interaction.

## IV. Sources of Error

The DALK procedure requires great precision, with less than 50 *μ*m on average separating a successful pneumodissection from a failure [35]. There are several sources of error which affect needle insertion performance. As the needle is inserted along its axial direction, both the cutting force at the needle tip required to separate the cornea tissue and the friction along the needle shaft are difficult to model. When the needle is pitched or translated along its lateral direction, it will pull against the cornea tissue, both deforming the cornea and applying torque to the needle. These forces can cause the needle shaft and holder to flex independently from the robot end-effector, accentuating the non-rigid relationship between the end-effector and needle. The deformation induced in the cornea also complicates planning by effectively creating a moving target. We have also observed that even after the robot has stopped moving, the needle and cornea may continue to move due to residual tension in the needle shaft and cornea.

Limitations of the perception system can also create error. The RMS tracking error for the needle tip position and orientation is typically around 12 *μ*m and 0.5° respectively, but failures of the ICP algorithm can occasionally lead to much larger errors or cause the needle tracking to fail entirely. Error in calibration and actuation give the robot a total repeatability of 25.4 *μ*m [13].

As shown in Fig. 2, the opaque needle shaft creates a shadow which makes segmentation of the posterior cornea surface beneath the shaft difficult and prone to error. This is complicated by the fact that as the needle shaft is inserted, the cornea tissue is displaced, causing the posterior cornea surface to deform downward. This deformation occurs within the needle’s shadow and makes it difficult to judge the needle’s depth in real-time.

Finally, there are also anatomical differences between individual corneas, due to factors such as the patient’s age, medical history, and in the case of *ex vivo* corneas, tissue preservation time. The cornea thickness varies, and we have observed that some corneas are more resistant to cutting than others. There is also variation in the cornea optical properties, and as a result we have observed that some corneas produce needle tracking failures and surface segmentation failures more frequently than others.

## V. Data-Driven Modelling of Needle-Tissue Interaction

We train a data-driven model to predict the cornea-needle interaction, based on the features extracted by the perception system and the commanded robot arm movements. Formally, we consider a discrete-time sequence of features and control inputs, where $x_{t}$ and $u_{t}$ denote the features and control inputs at time t respectively and $x_{l:h}$ and $u_{l:h}$ denote the sequence of features and control inputs respectively between times l and h inclusive. The learning task is to predict $X_{t+1:t+H}$ given $X_{1:t}$ and $u_{1:t+H-1}$, for a prediction horizon H.

### A. Dynamic Model

**Fig. 4.**
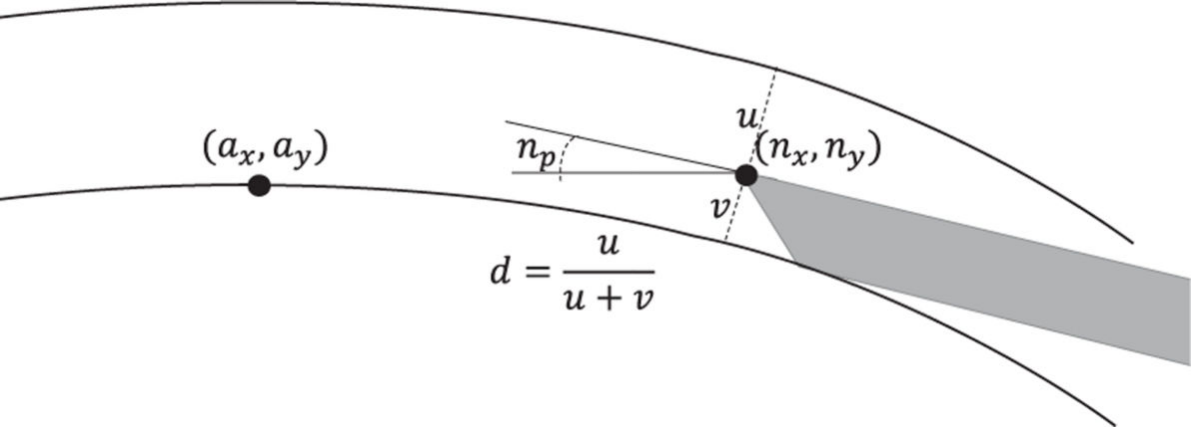
Selected state features include the 2D needle position $\left(n_{x},n_{y}\right)$, 2D apex position $\left(a_{x},a_{y}\right)$, needle pitch $n_{p}$, and needle depth d.

We select the dynamic model and features of the system state based on their relevance to planning (see Fig. 4) and model selection experiments in Section V-B. The needle state is represented by the 2D needle tip position in the OCT coordinate frame, denoted $\left(n_{x},n_{y}\right)$, and the needle pitch $n_{p}$. Since the needle predominantly moves within a plane and the OCT volume is re-sampled such that the z-axis is perpendicular to the needle shaft, it suffices to consider only two dimensions. We also include the 2D apex position in the OCT coordinate frame, denoted $\left(a_{x},a_{y}\right)$. Modelling apex movement allows the planner to account for cornea deformation as it guides the needle to its target position just above the cornea apex. Finally, we consider the needle depth ratio d. As shown in Fig. 4, the depth ratio is computed by finding the index-corrected ray normal to the anterior cornea surface which intersects the needle tip and then intersecting the ray with the posterior cornea surface. Modelling the depth ratio allows the planner to ensure that the needle is embedded in the cornea with sufficient depth and to accommodate variations in corneal thickness. The perception system also provides a segmentation of the cornea surface, but experiments below indicate that including cornea shape features reduces performance due to overfitting.

Control inputs are also modeled in the OCT coordinate frame. Prior to insertion, the system is calibrated using the method described in [13] to obtain a rigid transform between the robot end-effector and the needle tip. At each time step, forward kinematics calculates the *rigid needle tip position*, denoted $\left(r_{x},r_{y}\right)$, and the *rigid needle pitch*, denoted $r_{p}$. As previously noted, the rigid needle tip position will differ from the actual needle tip position due to the mechanical interaction of the needle and the cornea. We then choose our control inputs to be ${\left[u_{x}\right]}_{t}={\left[r_{x}\right]}_{t+1}-{\left[r_{x}\right]}_{t},{\left[u_{y}\right]}_{t}={\left[r_{y}\right]}_{t+1}-{\left[r_{y}\right]}_{t},{\left[u_{p}\right]}_{t}={\left[r_{p}\right]}_{t+1}-{\left[r_{p}\right]}_{t}$. Since movement is largely limited to the plane it suffices to consider only 2 dimensions. We define the control inputs in the OCT coordinate frame rather than the robot workspace and use the system calibration to convert to Cartesian robot movements. This approach makes the model invariant to variability in needle mounting.

Let *N* denote the number of insertions in the training set and let $x_{t}^{\left(i\right)}={\left[n_{x}\;n_{y}\;n_{p}\;a_{x}\;a_{y}\;d\right]}^{T}$ and $u_{t}^{\left(i\right)}={\left[u_{x}\;u_{y}\;u_{p}\right]}^{T}$ denote the features and controls respectively at the t-th timestep of the i-th insertion. Let $T_{i}$ denote the i-th insertion length. We model the cornea-needle interaction using a linear autoregressive model with an exogenous variable (ARX), which models the dynamics as a linear function of a fixed-size window of the feature and control history. ARX has a long history of use in time-series prediction and control problems [36] and is straightforward to use in MPC. In contrast to nonlinear models such as neural networks, trajectory optimization with an ARX model can be formulated as a quadratic program (QP), which can be optimized quickly and reliably. Specifically, ARX predicts ${\hat{x}}_{t+1}=A_{k}\Theta_{t}$ where

$$\Theta_{t}={\left[x_{t}x_{t-1}\ldots x_{t-k+1}u_{t}u_{t-1}\ldots u_{t-k+1}1\right]}^{T},$$

$A_{k}$ is the learned parameter matrix, and k is a hyperparameter controlling the size of the history window, which is also known as the order of the model. Let $\Theta_{t}^{\left(i\right)}$ denote the feature vector at the t-th time step of the i-th insertion. $A_{k}$ is learned by using linear least-squares regression to minimize the objective

$$\sum_{i=1}^{N}\sum_{t=1}^{T_{i}-1}{\left\|A_{k}\Theta_{t}^{\left(i\right)}-x_{t+1}^{\left(i\right)}\right\|}_{2}^{2},$$

Where $\|\cdot {\|}_{2}$ denotes the L2-norm. Since this ARX model considers features of the needle, depth, and apex, we refer it as the *ARXk-NDA* model. In order to make predictions within the first k time steps of an insertion, we also train separate ARX models with orders 1≤h≤k. We normalize the OCT coordinate frame by applying a translation so that the initial cornea apex position is at the origin. This makes the model invariant to translations of the cornea with respect to the OCT scanner.

### B. Training and Model Selection

**Fig. 5.**
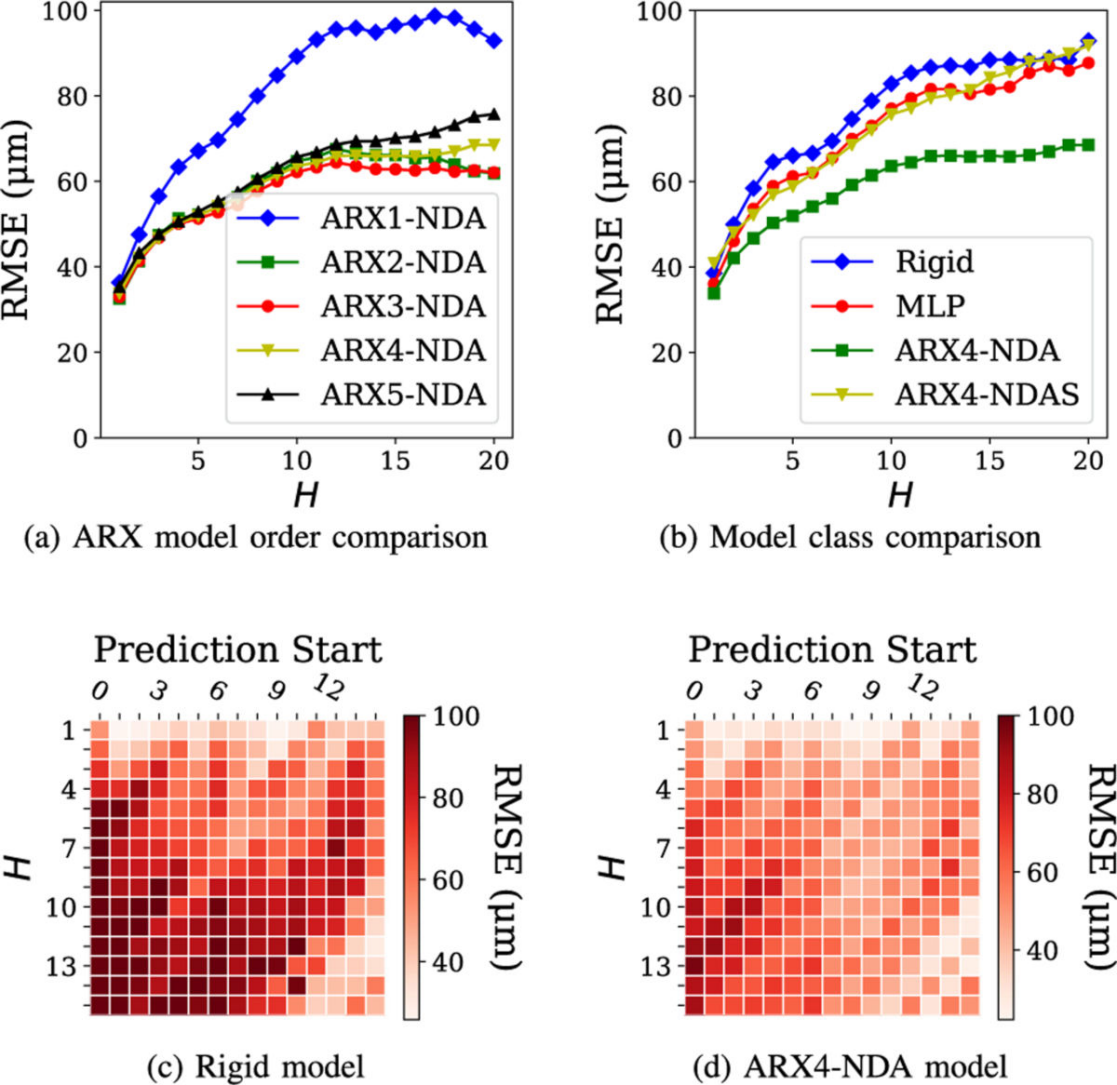
Comparison of several models in needle tip $\left(n_{x},n_{y}\right)$ prediction accuracy. Heatmaps in (c,d) evaluate model accuracy at varying prediction horizons H and prediction starting timesteps.

Using a dataset of 38 insertions from Draelos *et al.* [13], we combined 19 randomly selected insertions with 5 additional insertions taken from preliminary *ex vivo* experiments as a training set, and used the remaining 19 from Draelos *et al.* [13] for validation. Altogether, this yields 771 time steps in the training set and 545 time steps in the validation set. We compare the prediction accuracy of several models on the validation set at varying prediction horizons. In Fig. 5(a), we evaluate the impact of model order on the prediction accuracy of the ARXk-NDA model. We observe that at k=1, the model prediction accuracy is significantly worse, while among 2≤k≤5 the impact of model order is minor, only becoming apparent at the longest horizons. In Fig. 5(b) we compare ARXk-NDA to several other model classes, using k=4 as a representative example. We compare against the rigid model and two other data-driven models. The multi-layer perceptron (MLP) model operates over the same feature space as the ARXk-NDA models, and uses hyperparameters automatically selected by AutoMPC [37]. The ARX4-NDAS model expands the feature space to include points sampled along the top and bottom cornea sufaces. For each surface, 10 points are sampled at even intervals in the x-axis. We find that the ARX4-NDA model significantly outperforms each of these alternatives at most prediction horizons. A key property of the ARX model is that it considers a window of history, whereas the rigid and MLP models only use the most recent observation. The superior prediction accuracy of ARX suggests that historical observations and controls are very useful for predicting the needle-tissue interaction. On the other hand, the ARX4-NDAS model suffers from overfitting due to its larger feature space.

Figs. 5(c) and (d) evaluate the rigid and ARX4-NDA models respectively at varying prediction horizons and varying starting points in the insertion. We observe that the performance of the rigid model varies with the insertion starting point, with particularly high error occurring when prediction begins from the first few time steps. Though similar trends do hold for the ARX model, it does consistently outperform the rigid model at more than 90% of all starting points and prediction horizons.

## VI. Needle Advancement MPC

**Fig. 6.**
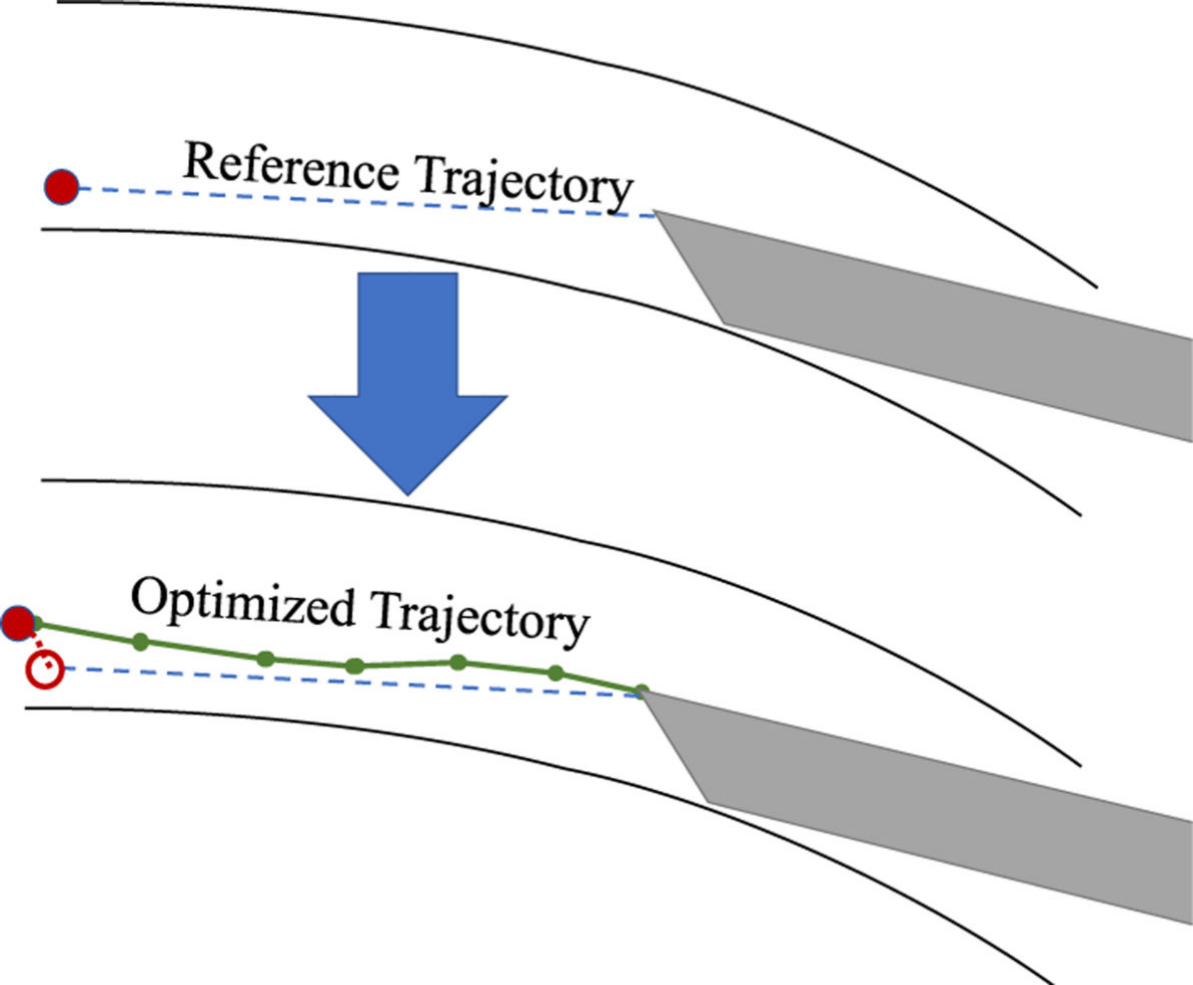
The needle advancement MPC begins by constructing a straight-line reference trajectory between the needle tip and goal, then optimizes a trajectory to track it, while predicting needle and apex motion.

Using the data-driven model of cornea-needle interaction, we design a model predictive controller (MPC) to control the needle advancement phase. At a high level, the controller begins by constructing as a reference trajectory a straight line path between the needle tip and the goal position (see Fig. 6). The controller then optimizes for a trajectory consistent with the data-driven dynamics model which closely tracks the reference trajectory. The optimized trajectory is penalized for deviations from the reference trajectory at both intermediary states and the terminal state.

### A. MPC Optimization

During the advancement phase, the controller is triggered whenever a new OCT volume is acquired and processed, which normally occurs every~0.65s. At this time, we observe the state $\left(n_{x},\;n_{y},\;n_{p},\;a_{x},\;a_{y},\;d\right)$. The needle starting point is adjusted to account for the expected time for planning, which is denoted $t_{\text{lag}}$. This is computed as $s_{x}=n_{x}+t_{\text{lag}}v_{x},\;s_{y}=n_{y}+t_{\text{lag}}v_{y}$, and $s_{p}=n_{p}+t_{\text{lag}}v_{p}$, where $v_{x},\;v_{y}$, and $v_{p}$ denote the current commanded needle tip velocities in x, y, and pitch respectively. The initial state for planning is $x_{\text{init}}={\left[s_{x}\;s_{y}\;s_{p}\;a_{x}\;a_{y}\;d\right]}^{T}$. The goal needle tip position is computed by adding a constant offset to the current apex position, $g_{x}=a_{x}+x_{\text{off}}$ and $g_{y}=a_{y}+y_{\text{off}}$. The goal needle pitch is horizontal $g_{p}=0$ and the goal depth is set to $g_{d}=90\%$. Finally, in order to limit cornea deformation, the goal apex is the same as the current apex position. Thus, the goal state is $x_{\text{goal}}={\left[g_{x}\;g_{y}\;g_{p}\;a_{x}\;a_{y}\;g_{d}\right]}^{T}$.

A reference trajectory is constructed for all state dimensions. The length of the reference trajectory is determined by the horizontal distance between the start position $s_{x}$ and the goal position $g_{x}$ as well as the desired *x*-axis speed $v_{x}^{\text{ref}}$. We have $m=\left\lfloor \left|s_{x}-g_{x}\right|/v_{x}^{\text{ref }}\right\rfloor$. The state reference trajectory is computed by linearly nterpolating between $x_{\text{init}}$ and $x_{\text{goal}}$. That is, the i-th step of the reference trajectory is given by $x_{i}^{\text{ref}}=\frac{i}{m}x_{\text{goal}}+\frac{m-i}{m}x_{\text{init}}$ for 0≤i≤m. The reference control trajectory is constructed as the sequence of controls needed to achieve the reference state trajectory assuming the rigid dynamics model. That is, for the *x*-axis, we have ${\left[u_{x}^{\text{ref}}\right]}_{i}={\left[n_{x}^{\text{ref}}\right]}_{i+1}-{\left[n_{x}^{\text{ref}}\right]}_{i}$ for 0≤i≤m and $u_{y}^{\text{ref}}$ and $u_{p}^{\text{ref}}$ are defined similarly.

Next, we optimize for a state and control trajectory which tracks the reference trajectory and is consistent with the data-driven dynamics model. This is done by constructing and solving a quadratic program (QP). Although the reference trajectory is m steps long, in order to account for movement of the needle after the robot has stopped moving, we solve for a longer m+l+1 state trajectory with the final l steps having zero control input. The quadratic program is given by

$$\min_{x_{0:m+l},u_{0:m-1}}\left(\sum_{i=1}^{m}{\bar{x}}_{i}^{T}Q{\bar{x}}_{i}+{\bar{u}}_{i}^{T}R{\bar{u}}_{i}\right)+{\tilde{x}}_{m}^{T}F{\tilde{x}}_{m}\;\;+{\tilde{x}}_{m+l}^{T}G{\tilde{x}}_{m+l}$$

$$\text{s.t.}\;x_{0}=x_{\text{init}}$$

$$\;x_{i}=A_{h}{\left[x_{i-1}\ldots x_{i-k}u_{i-1}\ldots u_{i-k}1\right]}^{T}$$

$$\text{where}\;{\bar{x}}_{i}=x_{i}-x_{i}^{\text{ref}}\;{\bar{u}}_{i}=u_{i}-u_{i}^{\text{ref}}$$

$$\;{\tilde{x}}_{i}={\left[{\left[n_{x}\right]}_{i}-{\left[g_{x}\right]}_{i}\;{\left[n_{y}\right]}_{i}-{\left[g_{y}\right]}_{i}\;{\left[d\right]}_{i}-g_{d}\right]}^{T}$$

h=min(i,k).

Q, R, F, and G denote tune-able cost matrices. The QP is solved using the OSQP [38] solver.

To achieve good real-world performance, there are several additional practical considerations. To avoid large out-of-plane angular changes near the apex, replanning is stopped once the needle-apex horizontal distance is less than 0.5 mm and the last active plan is followed until completion. The controller must also be able to gracefully handle failures of perception, for which we use a simple outlier rejection scheme. When the observed needle position differs from the needle position predicted by the rigid model by more than a certain threshold, we assume a perception failure. In this case, the rigid model prediction is used to replace the observed needle position, and the observed needle depth is replaced by the observation at the previous time step. Outlier rejection does not apply to the apex observations which are less susceptible to perception failures.

### B. Cross-Simulation Controller Selection

The designer of a data-driven MPC must make a number of key choices, such as feature selection, model class selection, cost matrix tuning, and regularization. Each of these can have a significant impact on system performance, so the designer must weigh each option carefully. The gold standard would be to evaluate each design option with a sufficiently large sample size of physical experiments in order to achieve a reliable comparison. However in many applications, this approach would be prohibitively expensive and time-consuming for comparing more than handful of candidate designs. Instead, designers often use simulators as a proxy for physical experiments. This approach often suffers from the so-called *sim-to-real* gap, where inaccuracies in the simulation create bias in the estimation of MPC performance, a problem which can be especially significant in hard to model systems such as those involving deformable objects. Moreover, in a novel application, developing a high-quality simulator may be itself a more challenging problem than designing a data-driven MPC. We faced both of these challenges in the DALK task. Physical experiments are not only time and labor-intensive, but consume a limited supply of cornea tissue samples, and the interaction of the needle with deformable tissue is very hard to model in simulation.

Instead we propose *cross-simulation controller selection*. We randomly re-sample the training set for the planning model in order to obtain a simulation model training set, which is used to train an ARX simulation model. We then simulate the needle advancement MPC using the planning model in the controller and the simulation model in place of a simulator. This process is repeated, each time with a new simulation model created through random re-sampling. This technique allows us to assess how robust the MPC is to model uncertainty caused by the limited size of the training set, and we find it to be empirically a good predictor of real-world performance.

We use the cross-simulation technique to compare several candidate controllers, which mainly differ in the choice of planning model. For each controller, we run 100 trials, each with a different simulation model and initial configuration. Each simulation model is of the same class as the planning model, but uses a randomly bootstrapped training set. Each initial configuration is sampled randomly from real-world data. For each trial, we evaluate the final depth error $\left|d-g_{d}\right|$ and the final error in vertical position $\left|n_{y}-g_{y}\right|$. We present the summary statistics in Table I.

First, we evaluate the impact of ARX model order on controller performance. We consider orders 3≤k≤5 and denote the corresponding controllers ARX3-NDA-MPC, ARX4-NDAMPC, and ARX5-NDA-MPC. For brevity, we will drop the -MPC suffix when this does not create ambiguity. We find that of these three controllers, ARX4-NDA achieves the lowest error both in final depth and final vertical position, which leads us to choose k=4 as the model order. We note that this occurs in spite of the fact that the ARX3-NDA model had slightly better prediction accuracy, suggesting that prediction accuracy alone is not sufficient to evaluate the suitability of a model for control. Qualitatively, we observe that every controller produces some outliers with much higher depth and vertical error, creating relatively high standard deviations. Typically, this is caused by sudden deformations of the needle or apex in the last few steps of the simulation. When the model fails to predict these deformations, the needle cannot correct, so errors remain high.

In the interest of avoiding overfitting and unnecessary complexity in trajectory optimization, we next consider performing feature selection amongst ARX models. We propose several controllers which use *hybrid models*, where ARX is used to predict only some state dimensions, while the rigid model is used to predict others. For example, the *ARX4-DA-RN-MPC* controller uses the rigid model to predict the needle tip, but uses ARX for the apex and depth dimensions. The *ARX4-ND-RA-MPC* controller is similar, but uses the rigid model to predict the apex. Since the rigid model cannot be used to predict the needle depth, we cannot use the hybrid model to assess the impact of modelling the depth dimension. Instead, the *ARX4-NA-MPC* controller entirely removes the depth feature from the model, as well as the associated cost terms in the MPC formulation. For these comparisons, we always take the simulation model to be the ARX4-NDA, rather than choosing the same class as planning model. We find that while the ARX4-NA slightly outperforms ARX4-NDA in terms of vertical accuracy at 36.43 ± 5.2 *μ*m vs 40.4 ± 8.1 *μ*m, none of the three outperform ARX4-NDA in terms of depth error, which is the most clinically relevant factor. Thus, we choose to proceed with ARX4-NDA-MPC in the *ex vivo* experiments.

## VII. Ex Vivo Experiments

We evaluate our controller on human cadaver corneas in an *ex vivo* setting, simulating intraocular conditions using an artificial anterior chamber (Katena Products; Denville, NJ). This model has been well-validated in literature [8], [39], and we used only cornea samples with short preservation times suitable for transplantation, so the effects of post-mortem deterioration should be minimal. We used 6 corneas and performed 8 needle insertions per cornea, for a total of 48 trials. The baseline controller is the automatic line planner used in [13]. For each cornea, we randomly assigned half of the trials to the MPC and half to the baseline. Two of the MPC trials were excluded due to needle tracking failures in the final steps of the insertion which prevented accurate measurement of the final depth. We choose the target depth to be 90% and measure the actual final depth based on the needle and cornea segmentations given by the perception system (referred to as auto-graded depth). Table II summarizes the results.

**Fig. 7.**
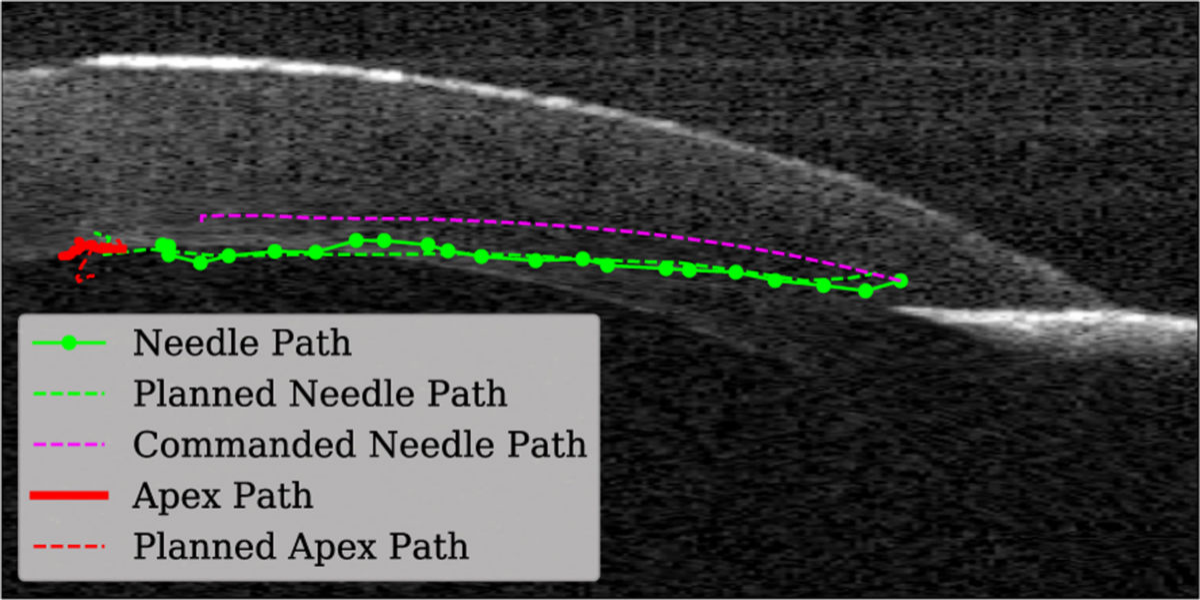
Example plan produced by MPC in testing.

We find that the MPC significantly (*p* < 0.05) out performs the baseline in terms of final depth error, and also outperforms the baseline in terms of final vertical error, though this result is not statistically significant due to the large variance in the baseline. This variance is caused by a number of factors, including perception noise, unpredictable needle and tissue deformation, and differences in cornea geometry and mechanical properties. We also note that under the baseline planner, the needle perforated the posterior cornea surface in 2 of 24 insertions, which would result in a clinical failure. No perforations were observed in the MPC insertions. Fig. 7 shows an example plan generated by the MPC in one of the *ex vivo* experiments. Qualitatively, we observe that the needle tracks the planned path well, and that the MPC correctly anticipated that the needle would take a lower path than the rigid model would have predicted.

**Fig. 8.**
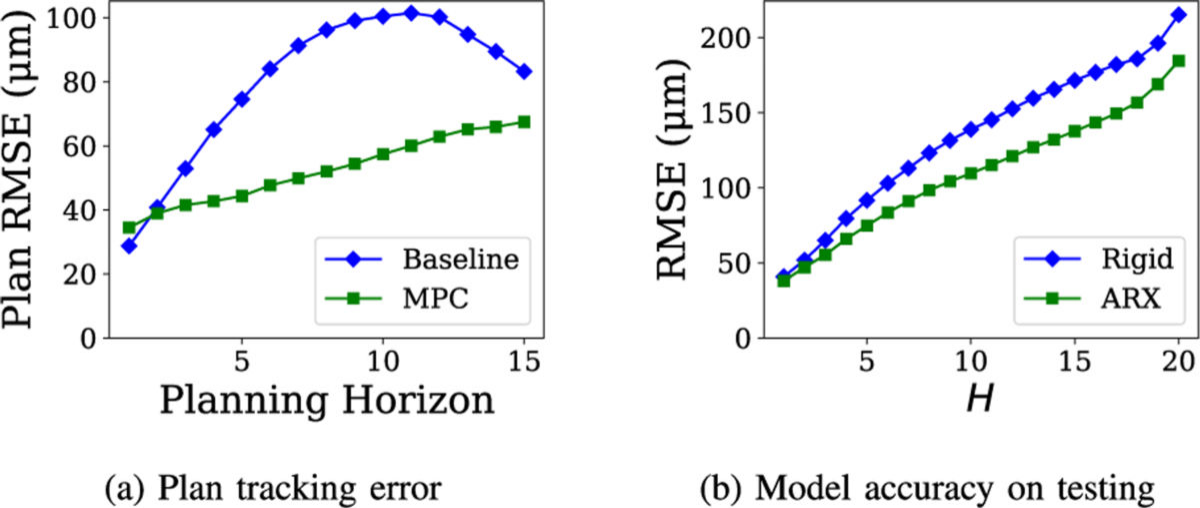
Analysis of the *ex vivo* experiments. 8(a) compares the plan tracking accuracy in the needle tip $\left(n_{x},n_{y}\right)$ dimension of the MPC vs the baseline planner; 8(b) evaluates model prediction accuracy in the needle tip $\left(n_{x},n_{y}\right)$ dimension on the *ex vivo* data.

To better understand the factors influencing performance in the *ex vivo* experiments, we perform *post hoc* analyses. First, we calculate the plan tracking error for both MPC and baseline. For a starting time t and a planning horizon $H_{\text{plan}}$, the plan tracking error is the minimum distance between planned path computed at time t and the actual position ${\left[\left(n_{x},\;n_{y}\right)\right]}_{t+H_{\text{plan}}}$. The plan tracking error is averaged over all insertions and starting points. In essence, this metric measures how accurately the needle reaches the positions intended by the planner. Fig. 8(a) compares the plan tracking errors of the MPC and baseline over varying planning horizons. We observe that while the MPC tracking error is higher than the baseline at the one-step planning horizon, the MPC exhibits lower tracking error for all longer horizons. This indicates that the MPC is superior to the baseline in producing realistic plans that can be followed by the needle. In the baseline trials, we frequently observe that the needle deflects significantly early in the insertions, but this deflection decreases as the needle is inserted deeper. As a result, the plan tracking accuracy of the baseline actually improves as the horizon increases from 10 to 15 time steps. Although the cause of this phenomenon is not known, it may be that as the needle is inserted, the tension created by needle deflection increases until some other part of the system begins to give, allowing the needle to return closer to its rigid position.

We also analyze model prediction accuracy on a testing set taken from the *ex vivo* experiments (Fig. 8(b)), comparing the ARX and rigid models. Both models perform worse on the testing set compared to the validation, but ARX still outperforms the rigid model at all prediction horizons. The difference in model accuracy between the validation and testing set may be partially explained by domain shift, since the MPC drives the system to a different distribution of states than the planners used in the training and validation sets.

Finally, we note that while the results presented here based on the auto-graded depth are significant, we also manually graded the depths to correct for perception errors and did not find a significant difference between the MPC and the baseline in terms of depth error (MPC 5.40 ± 1.96% vs Baseline 5.45 ± 3.11%). This indicates that the performance of the planner is constrained by limitations of the perception system. Improving the perception accuracy is outside the scope of this work, but an important area of future research.

## VIII. Discussion & Conclusion

In this paper, we demonstrate that data-driven techniques can accurately model the interaction between cannulation needle and cornea in the DALK procedure. We also demonstrate that the technique of cross-simulation controller selection can evaluate controller performance with offline data, and the results are predictive of real-world performance. The data-driven model predictive controller (MPC) developed using this approach enables the surgical robot to achieve superior accuracy in needle placement compared to the baseline. These improvements have the potential to signficantly improve the reliability and consistency of the DALK procedure. In future work, we hope to directly evaluate the impact data-driven MPC on the pneumodissection success rate, particularly when combined with an improved perception system.

## Figures and Tables

**TABLE I:** Results of Cross-Simulation Controller Selection. 100 Trials Are Run per Controller. We Report Final Error in Relative Depth and Vertical Position (Smaller Is Better), With ±95% CI

	Depth Error (%)		Vertical Error (μm)	
	*μ*	*σ*	*μ*	*σ*
**ARX3-NDA-MPC**	6.9± 1.0	5.1	41.3± 8.4	42.9
**ARX4-NDA-MPC**	6.1 ± 0.9	4.8	40.4± 8.1	41.3
**ARX5-NDA-MPC**	7.1 ± 1.2	6.2	65.9 ± 12.8	65.1
**ARX4-DA-RN-MPC**	41.5 ± 5.0	25.7	1,137.2 ± 90.1	459.9
**ARX4-ND-RA-MPC**	7.7 ± 1.0	4.9	50.6 ± 8.3	42.2
**ARX4-NA-MPC**	7.2 ± 0.9	4.5	36.3 ± 5.3	26.8

**TABLE II:** Results of *N* = 46 Insertions (22 MPC, 24 Baseline) on 6 *Ex Vivo* Corneas. We Report # of Perforations, Final Error in Relative Depth (auto-Graded), and Vertical Position (Smaller Is Better), With ±95% CI. P-Values Are From a Two-Sample T-Test

	Perforations	Depth Error (Auto) (%)		Vertical Error (μm)	
		*μ*	*σ*	*μ*	*σ*
**MPC**	0	3.96±1.48	3.35	43.29±15.78	35.60
**Baseline**	2	7.80±3.37	7.98	75.71±33.90	80.27
**P-Value**		0.039		0.082	
